# The effects of workplace friendship network centrality on deep acting

**DOI:** 10.3389/fpsyg.2023.1162086

**Published:** 2023-06-09

**Authors:** Na Yoon Kim, Hongseok Oh

**Affiliations:** ^1^Jay S. Sidhu School of Business and Leadership, Wilkes University, Wilkes-Barre, PA, United States; ^2^School of Business, Yonsei University, Seoul, Republic of Korea

**Keywords:** emotional labor, deep acting, friendship network centrality, positive affect, positive self-perception

## Abstract

We integrated social network theory with conservation of resource theory to predict that workplace friendship network centrality provides service employees with critical psychological resources that foster deep acting: positive affect and positive self-perception. In Study 1, we conducted a survey (*N* = 105) in a Korean banking firm, revealing that these resources mediate the relationship between workplace friendship network centrality and deep acting. Studies 2 and 3, both experimental studies, investigated the hypothesized causal relationships. In Study 2 (*N* = 151), we found that workplace friendship network centrality increases the intention toward deep acting. Further, Study 3 (*N* = 140) confirmed the direct effects of friendship network centrality on positive affect and self-perception. By providing insights into the structural antecedents of emotional labor, we inform managers in service organizations of the value of creating avenues for their employees to form and maintain friendships within the organization.

## 1. Introduction

Emotional labor, the management of feeling to create a publicly observable facial and bodily display (Hochschild, 1983), has become a key topic in organizational research due to the increasing demand to deliver high-quality customer service (Elfenbein, 2007; Grandey and Sayre, 2019). To enhance the quality of service outcomes, service employees are required to smile and be cheerful (Pugh, 2001). However, having to manage one’s emotions as part of a job can increase the level of stress and burnout, which impairs both the wellbeing of service employees and their long-term performance (Holman et al., 2002; Humphrey et al., 2015). To address the question of how the pain of emotional labor can be reduced while maintaining effective service performance, previous research has suggested that the emotional labor strategies that service employees adopt play a role in determining the outcomes of their emotional labor (e.g., Hülsheger and Schewe, 2011; Wiese et al., 2017). Two primary emotional labor strategies are surface acting and deep acting (Hochschild, 1983; Gabriel et al., 2015). Surface acting involves faking or expressing emotions that are not actually felt. In contrast, when service employees engage in deep acting, they modify their emotional state in an effort to experience the emotions they are expected to display. Deep acting has been found to lessen the psychological burden of service employees as their internal feelings and external emotional display are well-aligned (Grandey and Gabriel, 2015; Ma et al., 2023), and contribute to customers’ service quality perception (Groth et al., 2019). Building on this research, we aim to identify the factors that lead service employees to perform a more adaptive form of emotional labor, deep acting.

Over the past 30 years, much has been learned about the antecedents of emotional labor (Hülsheger and Schewe, 2011). However, as shown in the three-component model of emotional labor antecedents, outcomes, and moderators (Grandey and Gabriel, 2015), emotional labor research has primarily focused on two focal antecedents of emotional labor: personal characteristics and event characteristics. For example, personal characteristics, like agreeableness and dispositional affect, were found to be associated with deep acting (Grandey, 2000; Diefendorff et al., 2005), while customer mistreatment or injustice was associated with surface acting (Rupp et al., 2008, Sliter et al., 2013). Beyond these two levels, investigations into the impact of workplace social relationships on emotional labor have drawn from the notion that the social relationships employees form with other members of the organization affect various work outcomes (Heaphy and Dutton, 2008; Humphrey et al., 2016). For instance, research focusing on manager-employee relationships showed that abusive supervision is negatively related to deep acting (Wu and Hu, 2013), and a non-empathetic managerial approach toward employees is positively related to surface acting (Shani et al., 2014). Yet, relatively limited attention has been paid to the effects of service employees’ informal social relationships with coworkers on their deep acting toward customers, despite the relational nature of emotional labor itself.

In response to the call for more research on the antecedents of emotional labor (Grandey and Gabriel, 2015), the purpose of our paper is to investigate beyond the person- or event-level to elaborate on the social structural antecedents of deep acting. Drawing from the idea that an individual’s social network position represents the amount and kinds of psychological resources that the person gains from the social relationships (Balkundi and Kilduff, 2006; Brass, 2012), we combined a social network perspective with the resource-based view of emotional labor. We expect that service employees can gain crucial psychological resources that will help them perform deep acting. Unlike most prior emotional labor research describing positive social relationships as moderators that will reduce the detrimental impact of emotional labor on service employees (e.g., Grandey et al., 2012; McCance et al., 2013; Grandey and Gabriel, 2015), we suggest that service employees’ workplace friendship network, one of the positive relationships at work, is a social structural antecedent of deep acting toward customers. Further, we aim to demonstrate the mediation processes in which service employees’ workplace friendship network generates specific psychological resources—positive affect and self-perception—that lead to deep acting. We also tested the direct impact of friendship networks on both the resources and deep acting to examine the causality of influence among the variables in our research model.

## 2. Theory and hypotheses

### 2.1. A resource-based perspective of emotional labor and deep acting

A resource-based perspective of emotional labor draws from the conservation of resources theory to explain the depleting effects of emotional labor (e.g., Hobfoll, 2002; Brotheridge and Lee, 2003; van Gelderen et al., 2011). According to the conservation of resources theory, people seek to obtain, retain, and protect resources that are valued in their own right or that are valued because they act as conduits to the achievement or protection of valued resources (Hobfoll, 2001). When resources are threatened or lost, it increases stress and anxiety, causing exhaustion and other health issues. In addition, the theory emphasizes the effects of obtaining resources after substantial resource loss on one’s experience of stress. Those who successfully obtain and replenish psychological resources after substantial resource investment are less likely to suffer from negative outcomes than those who fail to gain resources (e.g., Wells et al., 1999; Billings et al., 2000). Service employees are particularly subject to a potentially vicious cycle of resource loss and gain as emotional labor uses up psychological resources and energy—requiring the constant acquisition of new resources to meet the demand of the job. Thus, it is critical to examine the sources of psychological resources and how it affects service employees’ emotional labor behavior. Indeed, if a service employee has ample energy and resources, they are more likely to effectively deal with their job by performing an active form of emotional labor, deep acting (Hobfoll, 2002; Liu et al., 2008; Huang et al., 2015). In contrast, those who lack resources will find it hard to handle the emotional demands of the job (Hobfoll, 1989), thereby engaging in the more defensive strategy of emotional labor, surface acting.

Relying on the reasoning that sufficient psychological resources will promote deep acting (Hülsheger and Schewe, 2011; Kammeyer-Mueller et al., 2013; Yoo and Arnold, 2014), we seek to understand whether critical resources stem from social relationships, enabling the service employees to perform deep acting, buffering them against the strain of emotional labor while enhancing service performance. Extant research on the antecedents of deep acting has focused on intrapersonal characteristics such as personality traits, motives, and abilities (Grandey and Gabriel, 2015). Service employees have been found to be more likely to perform deep acting when they have promotion and pleasure motives (Dahling and Johnson, 2010; Von Gilsa et al., 2014), higher emotional intelligence (Liu et al., 2008), or a strong prosocial orientation (Allen et al., 2010, Maneotis et al., 2014, Gabriel et al., 2015). Given that “resources” refer to anything that helps individuals attain their goals (Halbesleben et al., 2014), certain dispositions might benefit service employees as they respond to the emotional requirements of their job. However, those who lack beneficial dispositional characteristics can still obtain crucial resources from external sources—such as positive social relationships with coworkers.

In prior studies, positive social relationships have been often described as moderators that reduce the negative consequences of emotional labor (e.g., Grandey et al., 2012; McCance et al., 2013). As an example, a work context that is socially supportive may serve as a self-regulatory break for employees, and social sharing after a difficult customer exchange can help reduce feelings of anger (McCance et al., 2013). Additionally, a climate for authenticity among coworkers was found to lower burnout from emotional labor (Grandey et al., 2012). Although these findings related to the positive work environment are insightful, relatively little research is aimed at understanding the effects of social structure on emotional labor. There is a line of research suggesting that social structural characteristics (e.g., gender, race, or nation) affect how employees regulate their emotions (e.g., Hochschild, 1983; Grandey, 2000; Scott and Barnes, 2011; Kim et al., 2013). For example, racial minority members are more likely to use surface acting when they were the token member of their group (Kim et al., 2013). However, despite the relational nature of emotional labor, no research has investigated the impact of one’s social relationships at work from a social structural perspective. Thus, we address this gap by incorporating a social network perspective into emotional labor dynamics.

### 2.2. Workplace friendship network centrality: a social structural antecedent of deep acting

Social network theory focuses on social networks defined as the set of actors and the connections representing social relationships, or lack thereof, between the actors (Scott, 2011). According to this perspective, the pattern of individuals’ social networks determines their access to resources, social support, advice and information and, therefore, their interpretation and perceptions of organizational reality (Ibarra, 1993; Meyer, 1994). We maintain that the quality and quantity of psychological resources that service employees have depends on their social network position (e.g., Brass and Burkhardt, 1992; Burt et al., 2013). To service employees whose job requires constant regulation of emotion and self, friendship networks that are expressive and identity boosting may be particularly beneficial. Friendship networks developed with current coworkers are different from formal relationships like supervisor-subordinate relationships, as friendship at the workplace is not dictated by formal hierarchies (Adams and Blieszner, 1994). Friendships at the workplace can be characterized as informal, voluntary, expressive, horizontal, and symmetric social relationships (e.g., Kilduff, 1990; Morrison, 2002; Methot et al., 2016) that provide benefits such as emotional support (Karasek, 1979), development of positive identity (e.g., Sluss and Ashforth, 2007; Dutton et al., 2010), and work engagement and satisfaction (e.g., Chiaburu and Harrison, 2008).

Specifically, we theorize that service employees’ workplace friendship network centrality provides critical yet unique resources which facilitate deep acting. Workplace friendship network centrality represents the degree to which a given actor occupies a central position in the friendship network at work (Brass and Burkhardt, 1992). Having centrality in desirable social networks confers one a great amount of social power, information, prestige, and other important resources (Marsden, 1988; Perry-Smith, 2006). For example, those who are central within organizational friendship networks were found to interpret change as being within their control because their centrality provided them with essential confidence (Vardaman et al., 2012). Despite the potential benefits of having a central position, individuals cannot easily promote their centrality in pursuit of those resources because there are structural constraints coming from being embedded in a social network (Zhou et al., 2009). Having a centrality in a friendship network also requires a high level of out-degree centrality (i.e., the number of people the focal individual nominated as friends) and a high in-degree centrality (i.e., the number of people who nominated the focal individual as a close friend). In-degree centrality does not necessarily go up when an individual increases their out-degree centrality. Furthermore, some researchers have proposed that centrality in social networks such as work networks can drain one’s resources, as centrality in such a network may also increase the demands for work (Warr, 2002; Totterdell et al., 2004). However, we contend that the proportion of resources offset by centrality will be much lower in the workplace friendship network than in the work network, with the expectation that centrality in friendship network is less likely to cause high work demands (Totterdell et al., 2004). The main resources exchanged in the friendship network are social support, affection, and trust, not the workload or coercion.

Together, we suggest that having a central position within a workplace friendship network is a social structural antecedent of deep acting. Because one’s position in a social network determines the type and amount of resources that the individual can obtain, those who are central in the workplace friendship network can have more psychological resources. Consequently, they will be more likely to perform deep acting than service employees who have less central, if not peripheral, position in the workplace friendship network.

*Hypothesis 1*. Service employees’ workplace friendship network centrality is positively associated with their deep acting.

### 2.3. Positive affect as a mediator linking workplace friendship network centrality and deep acting

To examine the underlying mechanism through which service employees’ workplace friendship network centrality leads to deep acting, we focus on an informal social resource provided by workplace friendship networks—positive affect. According to the resource-based view of emotional labor, emotional labor constantly uses up one’s psychological resources and energy. Thus, the recovery of psychological resources is crucial for service employees to successfully carry out the demands of their job (Brotheridge and Grandey, 2002; Hennig-Thurau et al., 2006). We suggest that positive affect—the psychological experience of feeling active, enthusiastic, and positively energized (Watson et al., 1988; George, 1991)—serves as a psychological resource for deep acting. Research on the work-family spillover of feelings, has shown that an individual’s feelings in one social context can spill over to their emotional experience in other contexts (e.g., Judge and Ilies, 2004; Wagner et al., 2014). Thus, employees who feel positive due to their centrality in the workplace friendship network may also feel positive in their interactions with the customers. As such, two distinct social contexts are incorporated in the current paper: one involving the service employees’ friendships with other members of the organization and the other involving service employees’ relationships with customers. We illustrate how workplace friendship network centrality elicits positive affect and how positive affect, in turn, fosters deep acting toward customers.

There is suggestive evidence that supports the positive relationship between service employees’ friendship network centrality and positive affect. Empirical studies have demonstrated that intimate and frequent communications in friendship networks in general enable individuals to exchange their personal feelings and thoughts (Zech and Rimé, 2005; Tasselli et al., 2015). Such social sharing of emotions with friends improves individuals’ subjective feelings. Furthermore, friendships are one of the energizing relationships at work that can be a source of energy essential for performance and wellbeing (Heaphy and Dutton, 2008; Luke et al., 2020). Energizing relationships generate positive emotions, while de-energizing relationships produce negative emotions (Quinn and Dutton, 2005; Gerbasi et al., 2015). Thus, individuals who are central in the workplace friendship network would likely feel more positive than those who are less central. This is consistent with the positive spillover effects that denote that energizing relationships pass their positive experiences onto subsequent social interactions (Baker et al., 2003). Additionally, having a central position in the workplace friendship network will enhance the experiences of positive emotions at work because centrality provides individuals with more opportunities to control and access resources—spurring feelings of enthusiasm (e.g., Warr, 2002; Totterdell et al., 2004; Methot et al., 2016). Taken together, we predict that service employees who have established a central position in the friendship network at work are more likely to experience positive affect than those who have a peripheral position in the friendship network.

*Hypothesis 2a*: The more central position a service employee occupies in the workplace friendship network, the more likely the person experiences positive affect.

Positive affect represents the level of emotional energy an individual possesses (Hobfoll et al., 2000). To examine how positive affect facilitated by friendship network centrality promotes deep acting, we draw from the relationship between positive affect and individuals’ social motivation. Social psychologists have documented that positive affective states promote social tendency and acceptance (Clark and Isen, 1982; George, 1991; Carver et al., 1994; Griffith et al., 2021). That is, individuals tend to be more open to social events and tend to socialize more actively when they are in a positive affective state than when in a negative affective state. Some examples include how positive affect increases customer helping behavior (George, 1991), and how those who are feeling good tend to be more cooperative, even toward others who have no responsibility for their emotional state (e.g., Isen, 1987; Lawler, 1992). Provided that positive affect enhances social tendency, we propose that positive affect will promote service employees’ social motivation and acceptance toward customers, consequently leading them to actively experience the positive emotion they are required to express—deep acting. In addition, self-reinforcing motivation—the tendency of people in a good mood consciously try to reinforce good feelings while avoiding bad feelings (Clark and Isen, 1982; Dalal et al., 2009)—can explain how positive affect fosters deep acting. Because service employees in a positive affective state would want to reinforce their positive state, they would engage in deep acting during the service encounters. In contrast, those who are in a more negative affective state may not have the motivation to actively engage in deep acting. Therefore, we predict that positive affect facilitated by workplace friendship network centrality leads to deep acting.

*Hypothesis 2b*: The more positive affect a service employee experiences within an organization, the more likely the person engages in deep acting. *Hypothesis 2c*: Positive affect mediates the relationship between workplace friendship network centrality and deep acting.

### 2.4. Positive self-perception as a mediator linking workplace friendship network centrality and deep acting

In addition to the mediating role of positive affect, we predict that positive self-perception (Bem, 1967) would also mediate the relationship between friendship network centrality and deep acting. Psychologists and organizational researchers have investigated the essence of self and the effects of identity in various organizational contexts (Ashforth and Schinoff, 2016). The topic of self and identity is particularly important in the discussion of emotional labor because emotional labor is an act of regulating the presentation of self (Grandey et al., 2012). Prior research has found that the act of emotional labor is significantly related to how one perceives themselves on the job. Surface acting leaves little room for authentic self-expression, while deep acting permits authentic expression of self (Hochschild, 1983; Leidner, 1999). Further, service employees who strongly identified with their organizational roles found emotional labor a chance to act out their identity (Ashforth and Humphrey, 1993), as conforming to the emotional labor role is a way to feel authentic when their work roles are central in defining who they are. Such an association between one’s identity and emotional labor implies that positive self-perception may have an impact on deep acting, especially when emotional labor requires the expression of positive emotions.

According to the self-perception theory, people observe, recognize, and evaluate themselves as if they do it to others (Bem, 1967). Building on this idea, we explore how service employees get to perceive themselves more positively through workplace friendship network centrality as well as how positive self-perception further serves as a psychological resource that leads to deep acting. Self-knowledge mainly comes from social interactions with others (Mead, 1934/1981) and self-identity is negotiated through linked processes of self-exploration and the development of intimacy in relationships (Giddens, 1991). For example, Dutton et al. (2010) noted that work-based friendships and daily work interactions could make work a central domain for the construction of the self. This suggests that the characteristics of one’s social relationships, such as their network position at work, may serve as a basis of their self-perception. If a service employee is central in the workplace friendship network, that person may have more opportunities to acquire positive self-knowledge and to perceive oneself in a more positive manner than a service employee who has a peripheral position in the workplace friendship network. Indeed, those who occupy a central position in the friendship network often help others resolve relational conflicts and problems (Venkataramiani and Dalal, 2007). Thus, employees who are central in the workplace friendship network will have an enhanced sense of self as their positive identity is constantly affirmed by multiple friendship connections. Therefore, we hypothesize that service employees with a more central position in the workplace friendship network may perceive themselves more positively than those who are peripheral.

*Hypothesis 3a*: The more central position a service employee occupies in the workplace friendship network, the more likely the person perceives oneself in a positive way.

Uy et al. (2017) found that the negative effect of surface acting on emotional exhaustion can be mitigated by helping coworkers. This suggests that positive self-perception obtained from helping coworkers can protect service employees from the depleting effects of surface acting. To further examine the mediating effect of positive self-perception in the relationship between friendship network centrality and deep acting, we propose that positive self-perception can be a psychological resource that promotes deep acting which requires a motivation to try experiencing the emotion they need to display toward customers. One of the most influential motivations underlying the activation of self is the drive to engage in behaviors that will maximize their sense of cognitive balance or consistency (Korman, 1976; Wu et al., 2016). According to this consistency motivation, service employees who positively perceive their socio-emotional characteristics because of a central position in the friendship network would strive to continue activating their positive selves in customer interactions. To rephrase, service employees who view themselves as socially efficient and well-received will be more motivated to perform deep acting toward customers to maintain self-consistency. To do otherwise could create inconsistency in their sense of self. Furthermore, service employees who perceive themselves positively due to the centrality in the workplace friendship network likely have an increased sense of interpersonal competency and confidence—believing that they have the ability to carry out the demands of various social situations (Athay and Darley, 1982). Such positive self-perceptions of their social skills will foster self-efficacy in customer interactions, leading them to be more proactive in experiencing positive emotions. Therefore, an enhanced perception of their own socio-emotional self serves as a crucial psychological resource, allowing them to continuously enact their competent self through actively engaging in deep acting during interactions with customers. Taken together, we hypothesize that positive self-perception promoted by workplace friendship network centrality leads service employees to engage in deep acting.

*Hypothesis 3b*: The more positively a service employee perceives oneself within an organization, the more likely the person engages in deep acting.

*Hypothesis 3c*: Positive self-perception mediates the relationship between workplace friendship network centrality and deep acting.

We conducted three studies to investigate our hypotheses. Each study has distinct objectives. First, Study 1 examined in an actual work organization the mediating mechanisms in which an individual’s workplace friendship network centrality leads to deep acting through positive affect and self-perception. Study 2 focused on the causal relationship between workplace friendship network centrality and the behavioral intention toward deep acting in an experimental setting. Study 3, also an experimental study, assessed the causal relationship between workplace friendship network centrality and the two mediators, positive affect and self-perception.

## 3. Materials and methods

### 3.1. Study 1

We collected data from 10 branches of a Korean banking firm. One of the primary values and missions of this bank firm includes high-quality customer service and customer satisfaction. Each branch, ranging in size from 8 to 19 members, is considered to provide every member with equal chances of forming friendships. We used a roster method that has been widely used in research to examine the whole network properties (Oh et al., 2004). Completed responses were collected a week after the administration of the survey. Responses received from 105 respondents in nine branches were used for analysis, representing an 89% response rate. A branch where the response rate for the social network questionnaire was less than 80% was excluded (Mehra et al., 2001). The average age of respondents was 37 years; 46% of the respondents were men; 65% of the respondents were married; the average tenure at their branch was about 14 years; and the average hours spent in customer service per day was 6.7 h. Measurements that are originally in English were carefully translated into Korean, following the back-translation procedures (Brislin, 1986).

#### 3.1.1. Measures

##### 3.1.1.1. Friendship network centrality

We used a roster method to measure individuals’ friendship network centrality (Oh et al., 2004). Each respondent received a list of the names of all the members in their branch and responded to a question “To what extent did you go out with this person for social activities outside work such as going out to informal lunch, dinner, or drinks during the past 6 months?” They rated their relationships with each and every member on a five-point scale ranging from “not at all” (0) to “very much” (4). Using the responses on the rosters, we constructed value matrices of the nine friendship networks from nine branches. In the metrices, cell Xij represents i’s relation to j reported by i. Because friendships are characterized as reciprocated relationships in which i’s report of the relationship and that of j tend to be symmetric, we created the degree centrality of each respondent. Degree centrality captures both in-degree centrality (i.e., others reports of a given actor as a friend) and out-degree centrality (i.e., a given actor reports of others as friends), reflecting the symmetric nature of friendship networks. Furthermore, in the case of asymmetric reports in which two parties’ ratings on the relationship vary, we symmetrized the two ratings based on the lower rating to strictly capture the strength of their friendship. Through this process, the symmetrized degree centrality in the friendship network was calculated by UCINET (Borgatti et al., 2002).

##### 3.1.1.2. Positive affect

We used the PANAS scale (Watson and Tellegen, 1985) with an indication of a specific time frame of 1 month to capture the general affective state they experienced at work (e.g., Jovanović, 2015; Extremera and Rey, 2016). On the 10 items that measure positive affect such as active, strong, excited, and enthusiastic, respondents were asked to describe how they felt each emotion at work during the past month, using a five-point Likert-type scale ranging from “not at all” (1) to “very much” (5). (Cronbach α = 0.81). Despite some concerns on the self-reported measures of affect, we maintain that self-reports on one’s subjective feelings can provide valid and reliable results (Humrichouse et al., 2007; Extremera and Rey, 2016).

##### 3.1.1.3. Positive self-perception

Because we illuminate how one’s friendship network position determines the perception of their own socio-emotional characteristics, positive self-perception was measured by using the socio-emotional identity questionnaires (Milton and Westphal, 2005). We transformed those five items into the form of self-perception questionnaires (Schrauger, 1975). Sample items included “I am warm to others”; “I help the group lighten up when it comes tense”; and “I am understanding of others.” Respondents indicated the extent to which the statements represented themselves, using a five-point scale ranging from “strongly disagree” (1) to “strongly agree” (5) (Cronbach α = 0.75).

##### 3.1.1.4. Deep acting

We measured deep acting by using four items from Diefendorff et al. (2005). Sample items are “I try to actually experience the emotions that I must show to customers”; and “I work hard to feel the emotions that I need to show to customers.” Respondents indicated their responses on a five-point scale ranging from “strongly disagree” (1) to “strongly agree” (5) (Cronbach α = 0.83).

##### 3.1.1.5. Control variables

We controlled for demographic variables such as age, gender, and marital status that can affect emotional labor (Hochschild, 1983). Job tenure measured by months, daily service hours, and organizational rank have been also controlled according to the research findings on the impact of work experiences and service intensity on emotional labor (Rafaeli and Sutton, 1987).

#### 3.1.2. Common method bias

Common method biases can exist when data were collected from the same source because the respondents may alter their responses to maintain consistency among perceptions, attitudes, and attributions in their self-reported responses (Staw, 1975; Podsakoff et al., 2003). Given the nature of the key variables in the current research, we anticipate that common method bias might not be significantly severe. Specifically, in measuring the independent variable, workplace friendship network centrality, we used the degree centrality which is not solely determined by the self-reported perception of an individual’s own relationships with others (i.e., out-degree centrality) but also incorporated others’ evaluation of their relationship with the focal individual (i.e., in-degree centrality). Furthermore, we relied on the assumptions of traditional social network research such that individuals’ perceptions of their social structure are correlated with others’ perceptions of the same network and that the recollections of their relationships could accurately reflect their usual interactions (e.g., Romney et al., 1986; Freeman, 2004; Brands, 2013). Additionally, two mediators, positive affect and self-perception, denote how individuals subjectively feel and perceive themselves, which can be properly captured by self-reported measures. Nevertheless, to address the issue of potential common method variance as we collected self-reported responses from service employees, we conducted Harman’s one-factor test (Podsakoff and Organ, 1986; Podsakoff et al., 2003). We found that the largest factor did not account for the majority of the covariance in the variables (39%), which indicated that common method bias was not a severe threat in the current sample. We believe that results from this study cannot be attributed solely to the common method variance. However, because the strength of the observed relationships might have been affected by common method variance, future research may adopt procedural remedies such as the collection of data from different sources at different times.

#### 3.1.3. Results

Kaiser–Meyer–Olkin test (KMO) value was.879, and Bartlett test results [χ^2^ (171) = 987.61, *p* < 0.001] showed that there is no partial correlation amongst the variables. A principal component analysis using a varimax rotation revealed that questionnaire items have an internal validity. Three factors with an eigenvalue higher than 1.0 were extracted, accounting for 58.91% of the explained variance. Since factor loading values are higher than 0.4, no item was removed from the analysis.

**FIGURE 1:**
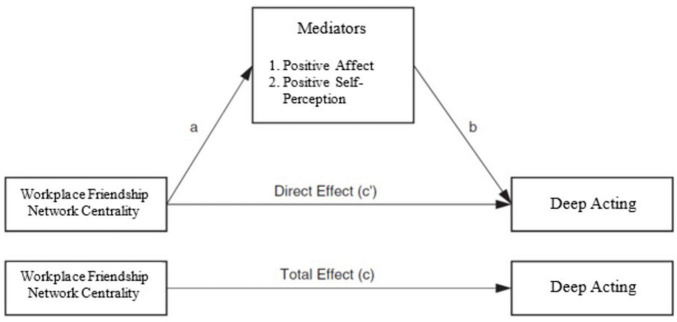
Research model illustrating our mediation hypotheses.

Table 1 presents the means, standard deviations, and correlations among the study variables. The results revealed that key variables including friendship network centrality, positive affect, positive self-perception, and deep acting are positively associated with one another. As shown in Figure 1, our model is a parallel mediation model that includes more than one mediator that are proposed to mediate the relationship between X and Y (Hayes, 2013). This model allows for a more complex examination of the process between X and Y. To test the mediation mechanism between workplace friendship network centrality and deep acting in which positive affect and self-perception serve as mediators, we used the PROCESS macro (Hayes, 2013). The total effect of the independent variable can be decomposed into direct and indirect effects (MacKinnon et al., 2002). The direct effect indicates the association between an independent variable and a dependent variable when the effects of mediators are controlled. The indirect effect represents the effect of a mediating variable in that relationship. To assess the indirect effects, the PROCESS macro uses bootstrapping which is a non-parametric resampling procedure. We tested the indirect effects with 5,000 bootstrap samples and a 95% confidence interval. An indirect effect is considered significant when zero is not within the range of the confidence interval.

**TABLE 1:** Means, SDs, and correlations among study variables.

Variable	1	2	3	4	5	6	7	8	9	10
1. Age	1	-	-0.293**	0.655**	0.915**	0.503**	0.01	0.243	0.271**	0.151
2. Rank	-	1	0.286**	-0.596**	-0.883**	-0.505**	-0.003	-0.253**	-0.287**	-0.163
3. Daily service hours	-	-	1	-0.124	-0.301**	-0.158	0.091	0.013	-0.021	-0.173
4. Marriage	-	-	-	1	0.161**	0.324**	-0.129	0.165	0.171	0.084
5. Tenure (months)	-	-	-	-	1	0.370**	0.005	0.226	0.288**	0.113
6. Gender	-	-	-	-	-	1	0.086	0.386**	0.224*	-0.026
7. Friendship network centrality	-	-	-	-	-	-	1	0.242**	0.205**	0.209*
8. Positive affect	-	-	-	-	-	-	-	1	0.520**	0.306**
9. Positive self-perception	-	-	-	-	-	-	-	-	1	0.344**
10. Deep acting	-	-	-	-	-	-	-	-	-	1
M	36.66	4.37	6.7	0.65	172.65	542	18.64	3.255	3.83	3.576
SD	8.545	1.756	1.599	0.48	124.57	0.5	14.13	0.709	0.563	0.612

*N* = 105, *p < 0.05, **p < 0.01, ***p < 0.001.

Hypothesis 1 predicted that service employees’ workplace friendship network centrality is positively associated with their deep acting. In support of Hypothesis 1, the total effect indicates that friendship network centrality is a significant predictor of deep acting, total effect = 0.010, SE = 0.004, 95% CI (0.0026, 0.0193). In order to test our mediational hypotheses predicted in Hypothesis 2 and 3, we conducted an analysis of the indirect effects. Results revealed a significant total indirect effect, total indirect effect = 0.0059, SE = 0.0022, 95% CI (0.0017, 0.0103), which suggests that decomposing the mediation into specific indirect effects is appropriate. We then tested Hypothesis 2 regarding the mediation process in which friendship network centrality fosters deep acting via positive affect. First, we found support for Hypothesis 2a which predicted that the more central position a service employee occupies in the workplace friendship network, the more that person is likely to experience positive affect (B = 0.017, *p* < 0.001). We also found support of Hypothesis 2b which predicted the positive association between positive affect and deep acting (B = 0.211, *p* < 0.05). Of more import, as predicted in Hypothesis 2c, we found a significant indirect effect of workplace friendship network on deep acting via positive affect, when controlling for the effect of self-perception (the other mediator), indirect effect = 0.0035, SE = 0.0020, 95% CI (0.0000, 0.0079). Next, we tested a set of Hypothesis 3 that concerned the relationship between workplace friendship network centrality and deep acting in which positive self-perception is a mediator. In support of Hypothesis 3a, we found that service employees who are central in the workplace friendship networks tend to perceive themselves in a more positive way (B = 0.010, *p* < 0.01). Hypothesis 3b that predicted the positive association between positive self-perception and deep acting was supported (B = 0.232, *p* < 0.05). Finally, as predicted in Hypothesis 3c, we found a significant indirect effect of workplace friendship network centrality on deep acting through positive self-perception when controlling for positive affect (the other mediator), indirect effect = 0.0024, SE = 0.0014, 95% CI (0.0000, 0.0054). As such, results from a parallel mediation analysis indicated that workplace friendship network centrality is indirectly related to deep acting through its relationship with positive affect and self-perception. As mentioned, the total effect was significant. However, the direct effect was no longer significant when those mediators are included in the model, suggesting a full mediation (B = 0.005, *p* > 0.1).

Table 2 summarized the indirect effect of workplace friendship network centrality on deep acting via positive affect and positive self-perception. Table 3 summarizes the direct and total effects of workplace friendship network centrality on deep acting. Although the findings of Study 1 proved that friendship network centrality provides service employees with critical psychological resources that enable deep acting, a direct causal relationship cannot be fully examined in a survey setting. Thus, to confirm there is a unique causal relationship between workplace friendship network centrality and the proposed psychological outcomes, we conducted two experimental studies on Amazon’s Mechanical Turk. First, Study 2 tested the direct effect of workplace friendship network centrality on deep acting predicted in Hypothesis 1. Study 3 then tested the causal relationships between friendship network centrality and the two mediators predicted in Hypothesis 2a and 3a. To emphasize, Study 2 and 3 are primarily focused on testing the causal relationships between the key variables, rather than the mediation process.

**TABLE 2:** Mediation analyses of the relation between workplace friendship network centrality and deep acting via positive affect and positive self-perception.

	Effect of FNC on mediators (a)		Effect of mediators on DA (b)		Indirect effect		
**Variable**	**B**	**SE**	**B**	**SE**	**B**	**SE**	**95% CI**
PA	0.017***	0.0045	0.211*	0.098	0.0035	0.002	(0.0000, 0.0079)
PSP	0.010**	0.0038	0.232*	0.115	0.0024	0.0014	(0.0000, 0.0054)

FNC, friendship network centrality; PA, positive affect; PSP, positive self-perception; DA, deep acting. *p<0.05, **p<0.01, ***p < 0.001.

**TABLE 3:** Direct and total effects of workplace friendship network centrality on deep acting.

	Direct effect of friendship network centrality (c’)			Total effect of friendship network centrality (c)		
**Variable**	**B**	**SE**	**95% CI**	**B**	**SE**	**95%CI**
Deep acting	0.0051	0.0042	(−0.0034, 0.0135)	0.01	0.0042	(0.0026, 0.0193)

### 3.2. Study 2

Participants completed a short online survey in exchange for $0.50. Among one hundred seventy responses collected from Amazon’s Mechanical Turk, 19 incomplete responses were excluded. As a result, 151 responses, 74 in high friendship network centrality and 77 in low friendship network centrality condition, were used in the analysis (Female, 28%; White, 90%, Latino, 7%, African American, 1.5%, Asian, 1.5%). Participants’ average age was 37 (range 21–60).

#### 3.2.1. Experimental manipulation

Respondents read that the study was about how individuals think and feel in a specific environment and that there were two phases in the study. They were randomly assigned to one of the experimental conditions, either high or low friendship network centrality. First, all respondents imagined that they were members of a hypothetical customer service department at a company. Depending on the experimental condition, they read about the different scenarios regarding their social structural position in the department. This task intended to elicit a specific perception of their position in a friendship network. No other information about the hypothetical organization was provided to control for the effects of any confounding variables.

Respondents in the high workplace friendship centrality condition read the following: “Please imagine that you are a member of a customer service department at a company. There, you have established friendships with many of the members, just like Jill in the graphic shown below. Take a moment to imagine what it would be like.”

Conversely, respondents in the low friendship centrality condition read the following: “Please imagine that you are a member of a customer service department at a company. There, you have established a friendship with only one other member, just like Lisa in the graphic shown below. Take a moment to imagine what it would be like.”

After the respondents completed this manipulation task, they responded to the questions that captured their behavioral intention to engage in deep acting.

#### 3.2.2. Measures

##### 3.2.2.1. Perceived workplace friendship network centrality

Respondents responded to two questions that were intended to affirm the effectiveness of the experimental manipulation in creating a variance in the perception of friendship network position between two experimental conditions. Respondents indicated their relationship with other members in the hypothetical customer service department based on what they read on the previous task, on a five-point Likert scale (1 = strongly disagree; 5 = strongly agree). The items are “I have many friends in this department.” and “I have established many friendships in this department” (Cronbach α = 0.92).

##### 3.2.2.2. Intention for deep acting

The respondents read the following questions before they indicated their behavioral intention for deep acting. “I asked you to imagine that you are a member of a customer service department. Your job here is to interact with customers to provide high-quality customer service. The company encourages each employee to engage in positive customer interactions and show positive emotions toward customers. How would you perform your job in this department?” Then, they indicated their thoughts on the four items that measured their intention for deep acting (Diefendorff et al., 2005), using a five-point scale ranging from “strongly disagree” (1) to “strongly agree” (5). Example items are “I would try to actually experience the emotions that I must show to customers”; and “I would work hard to feel the emotions that I need to show to customers” (Cronbach α = 0.89).

#### 3.2.3. Results

An independent-sample *t*-test that was conducted to compare the perception of friendship network centrality in high vs. low friendship centrality conditions yielded a significant result, t(149) = 8.60, *p* = 0.000. Respondents in the high friendship centrality condition reported a greater level of friendship network centrality perception (M = 4.16, SD = 0.90) than those in the low centrality condition did (M = 2.42, SD = 1.49). This indicated that the experimental manipulation successfully shaped the respondents’ perception of their friendship network centrality in the hypothetical customer service department. More importantly, in support of Hypothesis 1, we found that those in the high centrality condition had greater intention to engage in deep acting (M = 4.09, SD = 0.81) than those in the low friendship centrality condition (M = 3.70, SD = 1.07), t(149) = 2.53, *p* = 0.012. Although the respondents only imagined the situation in a hypothetical setting, the result provided stronger evidence of the causality between workplace friendship network centrality and deep acting. Imagining having a central position in a workplace friendship network increased their intention for deep acting. Developing on the findings of Studies 1 and 2, we conducted Study 3 to affirm the causal relationships between friendship network centrality and the two mediators, positive affect and self-perception.

### 3.3. Study 3

Participants completed a short online survey in exchange for $0.50. Among one hundred fifty responses collected from MTurk, 10 incomplete responses were excluded. As a result, 140 responses, 70 in high friendship network centrality and 70 in low friendship network centrality condition, were used in the analysis (Female, 34%; White, 91%, Latino, 6%, African American, 1.4%, Asian, 1.4%). Participants’ average age was 37 (ranging from 22 to 64).

#### 3.3.1. Experimental manipulation

We used the same experimental process as in Study 2 except that participants indicated their affective states and perception of themselves in Study 3. Like in Study 2, respondents in the high workplace friendship network centrality condition read the following: “Please imagine that you are a member of a customer service department at a company. There, you have established friendships with many of the members, just like Jill in the graphic shown below. Take a moment to imagine what it would be like.” Conversely, respondents in the low friendship centrality condition read the following: “Please imagine that you are a member of a customer service department at a company. There, you have established a friendship with only one other member, just like Lisa in the graphic shown below. Take a moment to imagine what it would be like.” After the respondents completed this manipulation task, they responded to the questions that captured their perceived friendship network centrality, the degree of positive affect, and self-perception.

#### 3.3.2. Measures

##### 3.3.2.1. Perceived workplace friendship centrality

Two questions investigated the effectiveness of the experimental manipulation in creating a variance in the perception of friendship network position between two experimental conditions. Respondents indicated their relationship with other members in the hypothetical customer service department based on what they read on the previous task, on a five-point Likert scale (1 = strongly disagree; 5 = strongly agree). Example items are “I have many friends in this department.” and “I have established many friendships in this department” (Cronbach α = 0.91).

##### 3.3.2.2. Expected positive affect

Because the respondents imagined a hypothetical situation, we measured the emotions they expected to experience in the hypothetical department, using the PANAS scale (Watson and Tellegen, 1985). On the 10 items that measure positive affect such as active, strong, excited, and enthusiastic, respondents reported the extent to which they would experience each of the emotions in the hypothetical department (1 = extremely unlikely; 5 = extremely likely) (Cronbach α = 0.91).

##### 3.3.2.3. Expected positive self-perception

We measured expected positive self-perception by asking the respondents to indicate the extent to which the following statements represent themselves in this hypothetical department. Respondents rated themselves on the five items adopted from the socio-emotional identity questionnaires (Milton and Westphal, 2005) such as “I am warm to others”; “I help the group lighten up when it comes tense”; and “I am understanding of others” (1 = strongly disagree; 5 = strongly agree). (Cronbach α = 0.90).

#### 3.3.3. Results

An independent-sample *t*-test that was conducted to compare the perception of friendship network centrality in high vs. low friendship centrality conditions yielded a significant result, *t*(138) = 2.95, *p* = 0.004. Respondents in the high friendship centrality condition reported a greater level of friendship network centrality perception (*M* = 4.15, *SD* = 0.62) than those in the low centrality condition did (*M* = 3.69, *SD* = 1.15). It reaffirmed that the experimental manipulation successfully shaped the respondents’ perception of their friendship network centrality. The result also showed that those in the high centrality condition expected to experience a greater level of positive affect in the department (*M* = 4.14, *SD* = 0.49) than those in the low friendship centrality condition (*M* = 3.86, *SD* = 0.72), *t*(138) = 2.62, *p* = 0.010. The results from this experimental study provided evidence for the direct causal relationships predicted in Hypothesis 2a and 2b. In support of Hypothesis 2a, we found that the more central one has in a workplace friendship network, the more positive affect the person would experience in that context. Further, in support of Hypothesis 3a, those in the high friendship centrality condition reported a greater level of positive self-perception in the hypothetical department (*M* = 4.13, *SD* = 0.53) than those in the low friendship centrality condition (*M* = 3.83 *SD* = 0.78), *t*(138) = 2.63, *p* < 0.01. This indicates that respondents who imagined that they occupied a central position in a friendship network would be more likely to perceive themselves in a positive way. Although Study 3 tested the respondents’ expectations in a hypothetical department, findings documented the direct, positive effects of friendship network centrality on the psychological resources proposed in our research model—positive affect and self-perception. In other words, the results confirmed that there exist causal relationships between friendship network centrality and the two mediators, respectively.

## 4. Discussion

In support of our hypotheses, the results from our survey and two experimental studies showed that friendship network centrality promotes deep acting through the mediating effects of positive affect and self-perception. As predicted, friendship network centrality was found to be a social structural antecedent of deep acting that provides psychological resources to service employees in the form of positive affect and self-perception, which in turn lead to deep acting. Consistent with the resource-based perspective of emotional labor, service employees who have more psychological resources due to their centrality in the workplace friendship network would be more likely to try to experience the positive emotions they need to display toward customers than those who are peripheral in the friendship network.

### 4.1. Theoretical contributions

Our results contribute to theory and research on emotional labor in several ways. First, we broaden the theoretical model of emotional labor by combining a social network perspective with the resource-based view of emotional labor. Because psychological resources determine the emotional labor strategy that a service employee adopts and, therefore, the consequences of the emotional labor (Côté, 2005), it is crucial to identify the sources of psychological resources—the antecedents of emotional labor. However, as noted by Grandey and Gabriel (2015), more research is needed to expand the scope of the antecedents of emotional labor. They found the most commonly studied antecedents were confined to person-level traits and event-level mistreatment by customers, and further suspected that events with coworkers may affect the service employees’ motivation to use certain emotion regulation strategies. This idea is consistent with the affective events theory (Weiss and Cropanzano, 1996) which contends that affective experiences at work influence employees’ evaluative judgments about their jobs. Although the interaction with hostile customers has been studied as a relational determinant of emotional labor (e.g., Spencer and Rupp, 2009), no existing study on emotional labor, to our knowledge, elucidated service employees’ social relationships with coworkers as a relational antecedent of emotional labor. Rather, positive social contexts have been described as moderators that would reduce the detrimental effects of emotional labor (Duke et al., 2009). The current study is the first to provide empirical evidence for the effects of workplace friendship networks on deep acting as well as the mediating roles of specific psychological resources in the relation between friendship network centrality and deep acting. Given the relational nature of emotional labor, our investigation of a relational antecedent of emotional labor is important to the literature on emotional labor. Service employees’ social network position at the workplace will determine the type and amount of psychological resources and, therefore, the emotional labor strategy they adopt.

As such, our results suggest that further investigations of the social structural antecedents to emotional labor are needed. We recommend future research examine other social network characteristics that could determine the types and amount of resources service employees gain and their resulting emotional labor. For example, it is possible that a central employee who provides work advice to a large number of colleagues may experience a sense of mastery of their job, thus being more likely to display more effective emotional labor. Additionally, while we focused on emotional labor that requires displaying positive emotions toward customers in our focal research question, it would also be interesting to explore the impact of workplace friendship networks on the type of emotional labor that requires the display of negative emotions (e.g., Sutton, 1991). Because those who are central in the friendship network may have more positive feelings and self-perception, the expression of negative emotions could potentially be more laborious due to the dissonance between their positive psychological state and the negative state required by their job. Finally, future research may examine other psychological resources such as social support in exploring the impact of friendship network centrality on deep acting. It is possible that a service employee who receives social support from colleagues would be more likely to be socially supportive of the customers, and thus perform deep acting.

Second, we theoretically integrated the two different social relationships that have been often studied separately—within organizational social networks and emotional labor toward customers. On one hand, one of the primary topics in the research of organizational social networks is the effects of an individual’s social network position on their behaviors and interactions with other members of the organization (Burt et al., 2013). On the other hand, research on emotional labor mainly focused on employee-customer interactions (Grandey, 2000; Côté, 2005). Some research has suggested that emotional labor can broadly encompass emotion regulation efforts extended toward other members of the organization that one works with (e.g., Ozcelik, 2013; Trougakos et al., 2015). However, consistent with the classic conceptualization of emotional labor as emotion regulation enacted by service employees in their customer interactions for a wage (Hochschild, 1983), we maintain that emotional labor toward “customers” is distinct from a broader level of emotional regulation or impression management targeted at their colleagues (Grandey and Gabriel, 2015). Drawing from this distinction, we found a link between the two unique social contexts. Specifically, our analyses based on a social network theory revealed that an individual’s social network position in one social context could shape the way they interact with others in another social context. In an extension of the current research, future research could explore other social contexts in which the resource cycle caused by workplace friendship networks can have an impact. Wagner et al. (2014) found that day-to-day surface acting was associated with stress outcomes experienced at home: emotional exhaustion, work-to-family conflict, and insomnia. Service employees who obtain psychological resources from their workplace friendship network centrality may not only be able to perform deep acting but also have healthier family relationships than those who have lower workplace friendship network centrality.

Third, the current research offers a broader implication for the conservation of resource theory as we explored the occurrence of deep acting in detail from a resource-based perspective. Our findings showed that friendship with colleagues provides service employees with specific psychological resources such as positive affect and positive self-perception that will aid them to perform deep acting. Notably, the mediation through positive affect was especially profound. It is in line with research that showed the power of positive affect. Just like positive emotions can undo some harmful effects of negative emotion (Fredrickson et al., 2000; Fredrickson, 2001), or can improve one’s capacity and willingness for self-regulation (Tice et al., 2007), positive affect facilitated by friendship network centrality can effectively replenish service employees’ resources and counteract the exhaustive aspect of emotional labor.

### 4.2. Limitations and future research

Our study also has several limitations that point toward possible future research. To capture the direct causal relationships amongst key variables, we conducted two experimental studies (Study 2 and 3) and reported consistent findings across all of the studies. However, the mediation model tested in a cross-sectional survey design (Study 1) warrants caution in interpretation. Because there is a possibility of reverse causality, one can speculate that those who perform deep acting toward customers would have more positive feelings and self-perceptions in the workplace. As a result, they may be more likely to nurture friendships with colleagues than the service employees who perform surface acting. We encourage longitudinal research to replicate our findings. A longitudinal social network study in which emotional labor is measured several months following the measurement of workplace friendship network centrality could affirm the mediation process between the social network and emotional labor in an organizational setting. A longitudinal study could address the potential common method bias present in our current finding. Although common method variance may not be a serious concern in the current research, we cannot fully rule out the possibility of common method biases. Thus, we recommended methodological remedies, such as collecting responses from multiple sources, in order to improve the reliability and validity of the data in future research (Podsakoff et al., 2003).

Next, Previous research on informal socializing relationships in the United States (e.g., Ibarra, 1992, 1993; Mehra et al., 2001) suggests that these relationships are universally important. Our research maintains that friendship network centrality serves as social capital across cultures. Although it was not our main objective to explore cross-cultural comparisons, we found a consistent pattern across the respondents in Republic of Korea (Study 1) and the U.S. (Studies 2 and 3). However, this raises the concern of generalizability of the findings. The data in Study 1 were sampled from a Korean banking firm—a collectivistic context. Although collectivism might have been an influence on the formation of various social relationships (Lee et al., 2019) such as friendship networks and customer relationships, the current research is limited in providing insights into the influence of national culture on emotional labor. Future research could investigate the emotional labor dynamics proposed in this study in service organizations that have an individualistic context, instead. Specifically, would friendship network centrality function as a facilitator of psychological resources or an inhibitor in increasing the level of psychological burden of service employees? Similar findings to Studies 2 and 3 might be found in service organizations with individualism. However, maintenance of centrality in friendship networks in a less collectivistic context could also potentially be a source of psychological burden which can impair the motivation to actively engage in customer interactions.

## 5. Conclusion

Many empirical studies have demonstrated that deep acting is a more adaptive form of emotional labor, yet little is known about the social structural antecedents of deep acting. Acknowledging the unique impact of workplace social networks on work behaviors, we integrated a social network perspective and the resource-based perspective of emotional labor. Our findings showed that workplace friendship network centrality provides service employees with crucial psychological resources, such as positive affect and self-perception, that lead to deep acting. In other words, emotional labor that has been generally considered painful can be less painful, if not enjoyable, when the service employees can obtain psychological resources from a workplace friendship network. Our study contributes to the field of emotional labor by highlighting the importance of service employees’ expressive, informal social relationships with coworkers and the psychological resources stemming from those relationships. Now that the relationship between workplace friendship networks and emotional labor has been established, we encourage additional investigation of the social network properties that relate to emotional labor outcomes, as well as the exploration of additional psychological resources that contribute to service performance and the wellbeing of the service employees.

## Data availability statement

The raw data supporting the conclusions of this article will be made available by the authors, without undue reservation, upon request.

## Ethics statement

Ethical review and approval was not required for the study on human participants in accordance with the local legislation and institutional requirements. The patients/participants provided their written informed consent to participate in this study.

## Author contributions

NK and HO contributed to the conception, design of the study, and manuscript revision. NK collected data, performed the statistical analysis, and wrote the first draft of the manuscript. Both authors contributed to the article and approved the submitted version.
